# Supplementary material for: Comparison of Personal or Indoor PM2.5 Exposure Level to That of Outdoor: Over Four Seasons in Selected Urban, Industrial, and Rural Areas of South Korea: (K-IOP Study)
Source: Int J Environ Res Public Health. 2023 Aug 30;20(17):6684. doi: 10.3390/ijerph20176684 (PMC10487920; doi:10.3390/ijerph20176684)
Supplement: Supplementary file 1 [file ijerph-20-06684-s001.zip › ijerph-2551443-supplementary.pdf]

Comparison of personal or indoor PM2.5 exposure level to that of outdoor: over four seasons in selected urban, industrial, and rural areas of South Korea: (K-IOP study)

Chiyou Song[1] Chris Lim[2] Birhan Legese Gurmu[3] Mingi Kim[1] Sangoon Lee[1] Jinsoo Park[1] and Sungroul Kim[1,*]

[1] Department of ICT Environmental Health System, Graduate School Soonchunhyang University, Asan, Republic of Korea; chiyou1997 @gmail.com
[2] Department of Community, Environment, and Policy, Zuckerman College of Public Health, The University of Arizona, Tucson, Arizona, USA; chrislim@arizona.edu
[3] Department of Environmental Health Sciences, Graduate School Soonchunhyang University, Asan, Republic of Korea
*Correspondence: sungroul.kim@gmail.com.

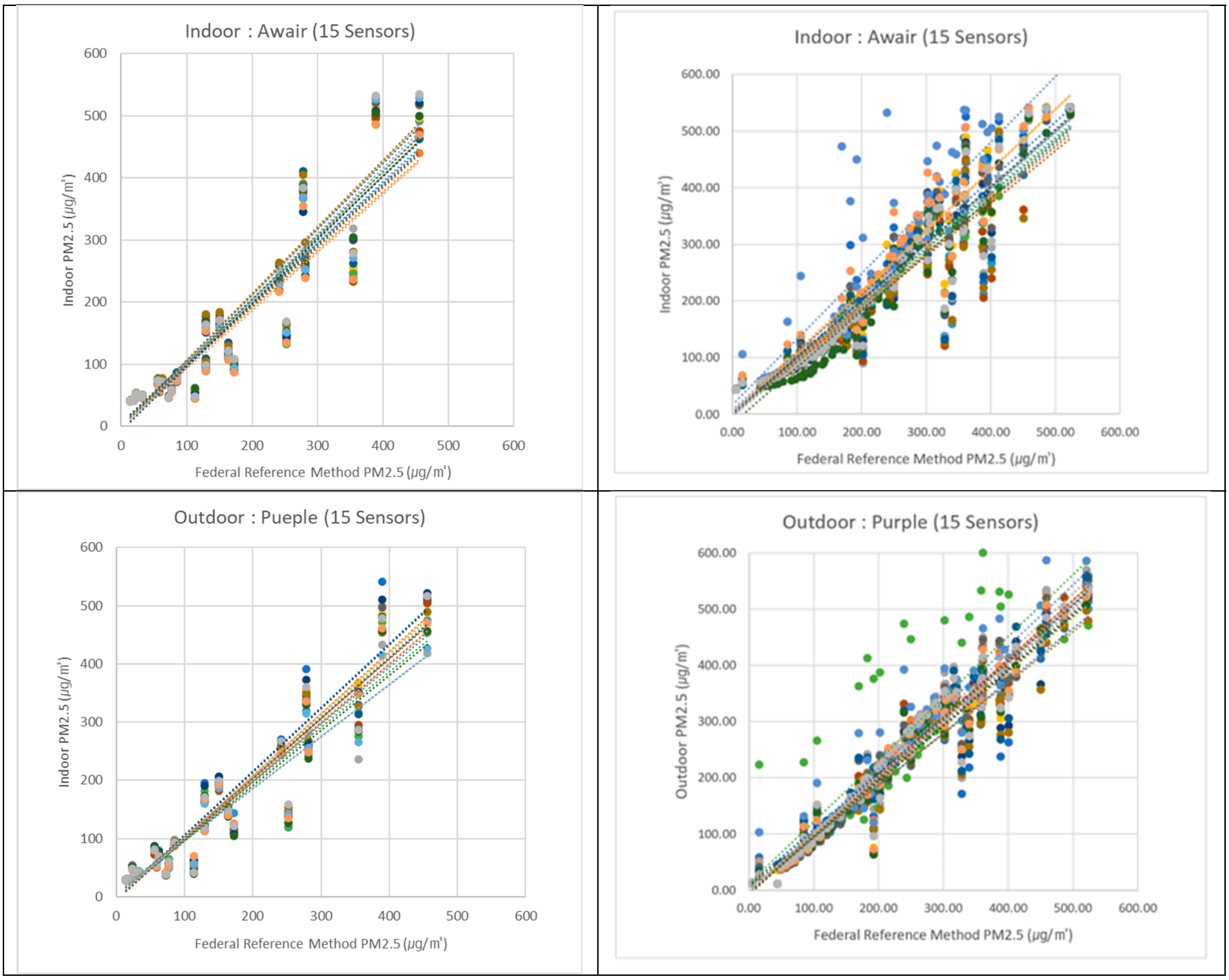

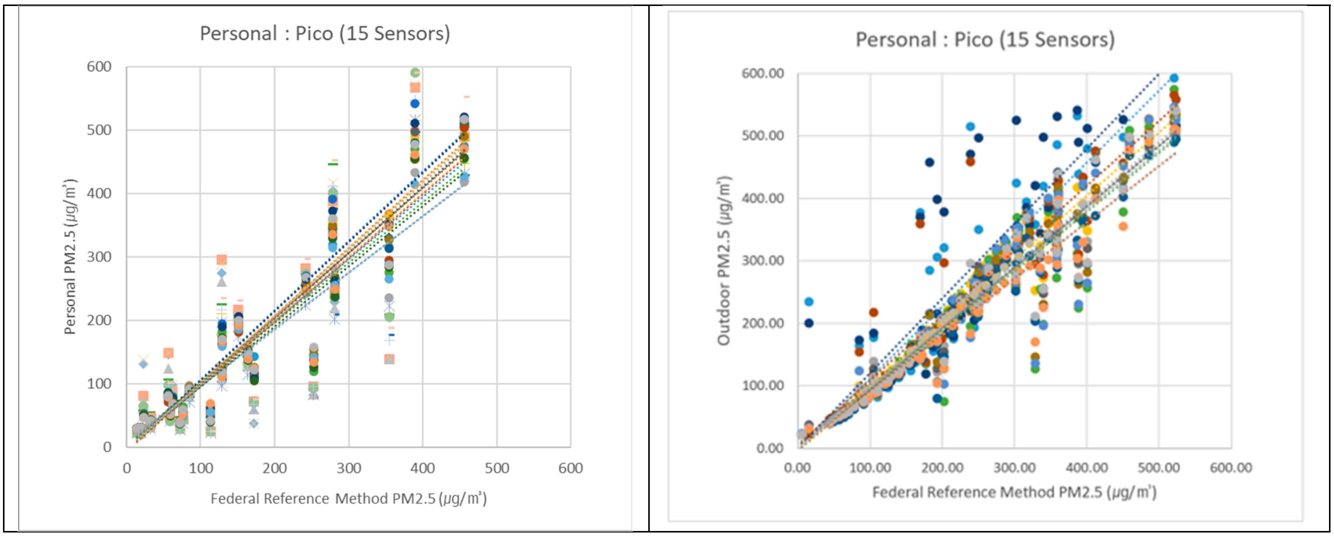

Figure S1. Scatter plots of distributions of PM2.5 obtained from indoor (Awair, U.S.A), outdoor (PurpleAir, U.S.A), and personal (Pico, Korea) sensors, compared to that of federal reference method (Grimm 11-D, German) : Experiment conducted at the living lab container of Soonchunghyang University South Korea: start of study (left), end of study (right).

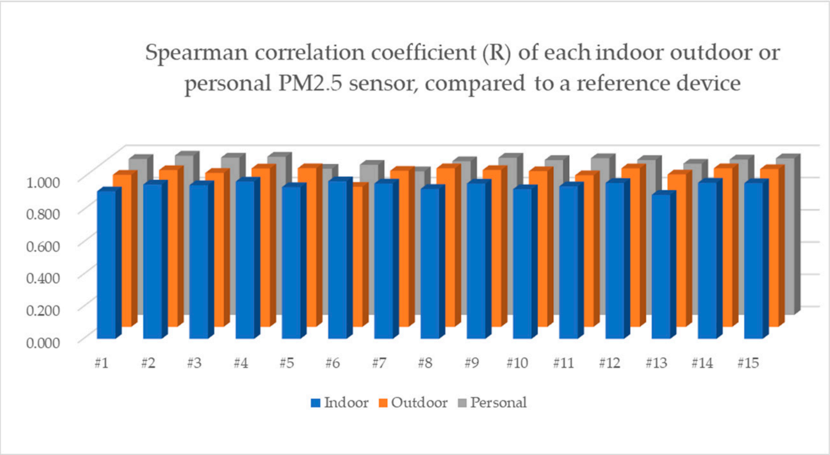

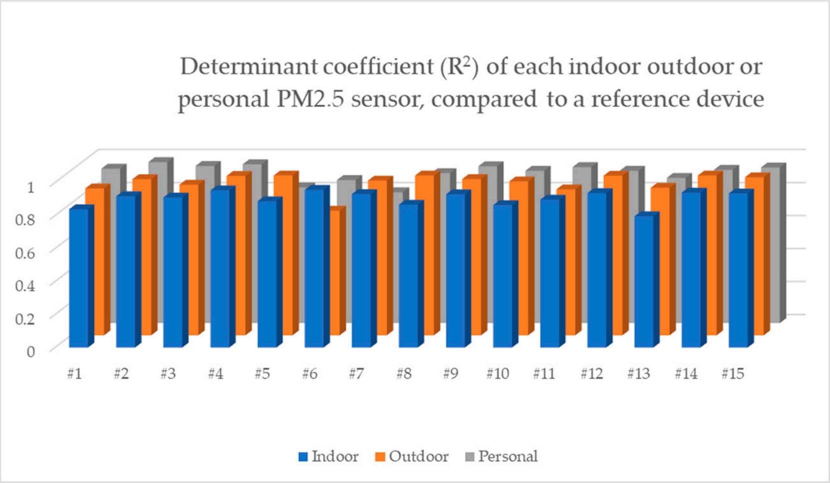

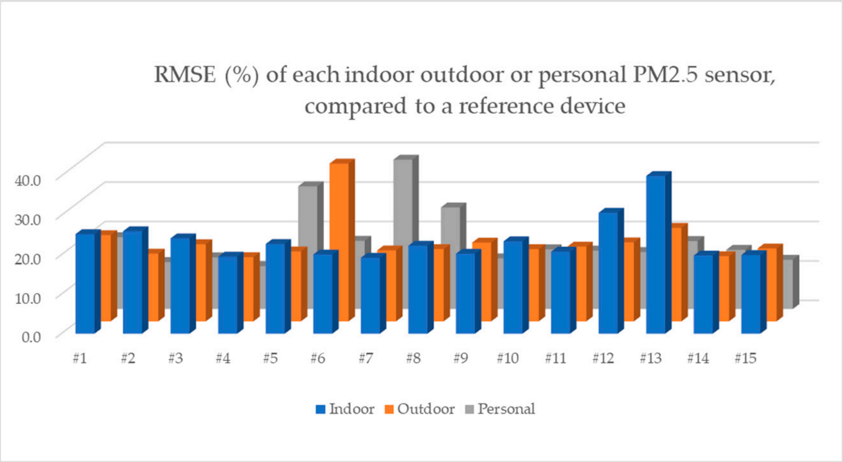

Figure S2. Levels of Spearman correlation coefficient (R), determinant coefficient (R^2) and root-mean-square-error RMSE obtained from indoor (Awair, U.S.A), outdoor (PurpleAir, U.S.A), and personal (Pico, Korea) sensors, compared to that of federal reference method (Grimm 11-D, German) : Experiment conducted at the living lab container of Soonchunghyang University South Korea: results from end of study. (RMSE (%) was calculated by applying U.S. EPA sensor validation guideline and modification of bias calculation procedure previous study reported by *Kim S, et al. (2019)* Evaluation of Performance of Inexpensive Laser Based $PM_{2.5}$ Sensor Monitors for Typical Indoor and Outdoor Hotspots of South Korea *Appl. Sci. 9*(9), 1947

Supplementary Table S1. Median (IQR) level of PM2.5, temperature, relative humidity, and pressure measured and national site and real-time sensors by season and area : Fall

	Fall			
	Urban	Industrial	Rural	P-value* (U:I:R)
$PM_{2.5}$_N (μg/m³)	17.0 (11.0-28.0)	18.0 (11.0-33.0)	15.0 (9.0-25.0)	<0.001
$PM_{2.5}$_O (μg/m³)	13.5 (7.8-23.9)	21.4 (12.3-38.5)	16.3 (8.7-31.3)	<0.001
$PM_{2.5}$_I (μg/m³)	6.2 (3.6-10.5)	5.9 (3.6-9.5)	6.5 (3.9-11.1)	<0.001
$PM_{2.5}$_P (μg/m³)	8.4 (6.1-12.6)	8.8 (6.5-13.5)	9.2 (6.5-15.8)	<0.001
Temp_O (℃)	17.8 (13.8-22.1)	10.2 (6.7-14.2)	9.3 (6.1-13.6)	<0.001
Humid_O (%)	34.2 (26.4-42.5)	40.8 (33.1-48.6)	47.6 (40.0-54.3)	<0.001
Press_O (hPA)	1021.9 (1015.7-1026.5)	1023.4 (1017.6-1027.4)	1024.8 (1018.9-1029.0)	<0.001
Temp_I (℃)	21.5 (19.1-23.3)	20.2 (18.6-22.3)	17.9 (15.4-20.8)	<0.001

Humid_I (%)	40.3<br>(33.6-48.9)	42.1<br>(34.0-51.2)	46.9<br>(42.0-52.2)	<0.001

* Kruskal-Wallis test

Supplementary Table S2. Median (IQR) level of PM2.5, temperature, relative humidity, and pressure measured and national site and real-time sensors by season and area : Winter

	Winter			
	Urban	Industrial	Rural	P-value* (U:I:R)
$PM_{2.5}$_N (μg/m³)	19.0<br>(13.0-32.0)	20.0<br>(13.0-38.0)	19.0<br>(13.0-40.0)	<0.001
$PM_{2.5}$_O (μg/m³)	19.6<br>(12.6-32.6)	23.1<br>(14.6-41.4)	19.7<br>(12.1-44.5)	<0.001
$PM_{2.5}$_I (μg/m³)	7.5<br>(4.4-13.5)	7.3<br>(4.8-12.6)	7.8<br>(4.5-15.7)	<0.001
$PM_{2.5}$_P (μg/m³)	8.8<br>(6.1-14.5)	9.4<br>(6.7-16.4)	10.1<br>(7.3-18.3)	<0.001
Temp_O (℃)	8.8<br>(2.3-14.4)	3.0<br>(0.6-7.0)	3.8<br>(0.6-8.2)	<0.001
Humid_O (%)	28.0<br>(21.5-35.8)	32.7<br>(26.9-38.9)	38.4<br>(31.2-45.7)	<0.001
Press_O (hPA)	1022.9<br>(1019.5-1026.6)	1024.1<br>(1020.7-1027.8)	1024.9<br>(1021.0-1029.0)	<0.001
Temp_I (℃)	22.9<br>(20.7-24.6)	20.1<br>(18.6-22.8)	19.0<br>(13.2-21.5)	<0.001
Humid_I (%)	30.0<br>(22.7-44.8)	32.1<br>(25.2-40.6)	33.3<br>(25.8-44.2)	<0.001

* Kruskal-Wallis test

Supplementary Table S3. Median (IQR) level of PM2.5, temperature, relative humidity, and pressure measured and national site and real-time sensors by season and area : Spring

	Spring			
	Urban	Industrial	Rural	P-value* (U:I:R)
$PM_{2.5}$_N (μg/m³)	16.0<br>(10.0-28.0)	18.0<br>(12.0-30.0)	16.0<br>(10.0-24.0)	<0.001
$PM_{2.5}$_O (μg/m³)	15.4<br>(8.7-29.5)	20.9<br>(11.8-36.8)	18.8<br>(11.3-30.2)	<0.001
$PM_{2.5}$_I (μg/m³)	6.6<br>(3.9-12.1)	7.4<br>(4.7-11.7)	7.2<br>(4.6-11.8)	<0.001
$PM_{2.5}$_P (μg/m³)	9.1<br>(6.5-15.3)	9.7<br>(7.0-14.4)	9.7<br>(7.4-15.2)	<0.001

Temp_O (℃)	19.5 (15.4-22.1)	15.1 (12.2-18.3)	13.4 (9.6-17.6)	<0.001
Humid_O (%)	31.7 (23.5-42.0)	39.2 (28.7-49.2)	47.1 (35.6-57.0)	<0.001
Press_O (hPA)	1020.6 (1016.4-1024.6)	1020.8 (1016.4-1025.0)	1022.7 (1017.2-1026.9)	<0.001
Temp_I (℃)	22.6 (20.0-24.4)	20.7 (19.1-22.6)	19.0 (15.6-21.4)	<0.001
Humid_I (%)	44.3 (36.2-53.0)	47.9 (39.3-57.0)	49.7 (40.9-58.6)	<0.001

* Kruskal-Wallis test

Supplementary Table S4. Median (IQR) level of PM2.5, temperature, relative humidity, and pressure measured and national site and real-time sensors by season and area : Summer

	Summer			
	Urban	Industrial	Rural	P-value* (U:I:R)
$PM_{2.5}$_N (㎍/㎥)	10.0 (5.0-20.0)	8.0 (4.0-18.0)	6.0 (3.0-12.0)	<0.001
$PM_{2.5}$_O (㎍/㎥)	10.1 (4.2-19.2)	10.5 (4.2-20.5)	8.1 (2.9-16.3)	<0.001
$PM_{2.5}$_I (㎍/㎥)	6.0 (3.2-10.8)	6.5 (3.8-11.1)	5.4 (3.3-9.0)	<0.001
$PM_{2.5}$_P (㎍/㎥)	8.8 (5.9-16.1)	7.9 (5.8-15.1)	7.1 (5.6-12.3)	<0.001
Temp_O (℃)	31.6 (29.9-33.3)	30.1 (28.4-31.9)	29.3 (27.6-31.6)	<0.001
Humid_O (%)	53.8 (47.9-58.7)	57.8 (51.6-63.1)	62.9 (54.6-69.0)	<0.001
Press_O (hPA)	1003.5 (1000.1-1007.0)	1004.0 (1000.8-1007.2)	1004.9 (1001.2-1008.2)	<0.001
Temp_I (℃)	27.8 (26.9-29.0)	27.6 (26.5-28.6)	27.0 (25.8-28.3)	<0.001
Humid_I (%)	72.7 (65.9-77.6)	73.6 (68.0-78.0)	75.3 (69.6-82.0)	<0.001

* Kruskal-Wallis test
